# Oral health changes during pregnancy and their association with stress and salivary cortisol

**DOI:** 10.1186/s12903-026-07957-9

**Published:** 2026-03-21

**Authors:** Nahla Hazem Ghanem, Radwa R. Hussein, Mustafa M. Abbas, Nevine H. Kheir El Din

**Affiliations:** 1 Department of Oral Medicine and Periodontology, Faculty of Dentistry, Ain Shams University, Cairo, Egypt; 2 Faculty of Medicine, Ain Shams University, Cairo, Egypt

**Keywords:** Oral changes, Pregnant women, Salivary changes, Pregnancy-related stress, Oral mucosal changes, Salivary cortisol

## Abstract

**Background:**

Pregnancy is often seen as a joyful and eagerly anticipated time in a woman’s life, yet it is not without its challenges, as it brings about various physical and hormonal changes. Oral changes are one of those changes that are frequently encountered during pregnancy. This necessitates enough research on the prevalence of those oral changes in different populations, their possible reasons, their association with pregnancy-related stress and the possibility of salivary cortisol changes influencing the oral cavity.

**Methods:**

A total of 160 participants were included for the present observational cross-sectional study (40 participants in each trimester of pregnancy, 1st, 2nd and 3rd, and 40 non-pregnant participants as a control group). Data about demographic characteristics (including age and parity), oral health (including hard tissue and soft tissue changes) and quality of life during pregnancy (including vomiting history and perceived stress) were recorded. Unstimulated salivary samples were collected via spitting method, for measurement of salivary cortisol and salivary pH.

**Results:**

Several oral findings, including gingivitis, soft‑tissue swellings, candidiasis, and dental erosion, were observed more frequently among pregnant participants compared with controls. Salivary cortisol levels were higher in pregnant groups, with the highest levels observed in the first trimester. Salivary cortisol level was found to be higher in the study groups than the control group, with the first trimester being the highest of all groups. Significant associations were identified between oral hygiene and caries incidence, vomiting history and certain oral mucosal changes, salivary cortisol levels and soft‑tissue lesions, and TPDS scores and soft‑tissue changes. These findings represent associations rather than causal relationships.

**Conclusion:**

Pregnancy was associated with a higher prevalence of specific oral and salivary findings, which showed correlations with psychosocial stress measures and salivary cortisol levels.

## Introduction

Pregnancy is considered a period of vulnerability due to the significant physiological changes that occur under hormonal influence. Vulnerable groups, including pregnant women, are generally more susceptible to health issues, including oral diseases [1]. The general health and well-being of pregnant women are closely linked to their perinatal oral health, which also plays a critical role in the health outcomes of their newborns [2]. Bacteria originating from infected periodontal tissues and their byproducts can reach the fetus-placenta unit, potentially leading to complications such as preterm birth, low birth weight, pre-eclampsia, or even stillbirth [3]. Additionally, gestational diabetes shares several risk factors with periodontal disease, including advanced maternal age, sedentary lifestyle, obesity, and poor dietary habits [4]. Furthermore, reducing cariogenic bacteria in the mother’s oral cavity may delay the colonization of Mutans streptococci in the infant, offering a protective effect [5].

Multiple factors contribute to the oral changes observed during pregnancy, including hormonal fluctuations, vomiting, dietary modifications, oral hygiene practices, and stress. Elevated levels of estrogen and progesterone during pregnancy facilitate the proliferation of periodontal pathogens, promoting gingivitis and periodontitis [6, 7]. Increased sugar cravings and inadequate oral hygiene further raise the risk of dental caries [8]. Erosion of teeth and structural changes in the oral mucosa are often associated with prolonged and severe pregnancy-induced vomiting [9]. Moreover, anxiety and depressive disorders, which are common during pregnancy, can cause long-term imbalance in homeostatic mediators, indirectly influencing oral health [10].

Several oral mucosal lesions are frequently encountered during pregnancy. Pyogenic granulomas, commonly known as pregnancy tumors, are prevalent and are linked to hormone-induced changes in vascular permeability [11]. Other less common but documented oral mucosal conditions include candidiasis, often associated with the acidic oral environment during pregnancy, cheek and lip biting due to stress and anxiety, and herpes labialis, which poses a risk of neonatal Human Simplex Virus (HSV) infection during birth [12–14].

Vomiting can be one of the indirect factors for the incidence of oral candidiasis during pregnancy through creating favorable conditions by the disruption of oral pH, mucosal damage and possible xerostomia due to dehydration.

Salivary analysis has emerged as a valuable tool for assessing physiological and pathological changes and for early disease detection [15]. However, findings regarding salivary changes during pregnancy remain inconsistent. While some studies suggest that pregnancy alters the quantity and quality of saliva, impacting periodontal and dental tissues, others report no significant changes [16]. Notably, salivary pH tends to decrease, contributing to periodontal issues [17]. Although salivary cortisol, a reliable indicator of serum cortisol and a recognized stress biomarker has been linked to increased dental biofilm and deteriorated gingival health in some studies [18].

Psychosocial stress during pregnancy, defined as the perception of unworthiness, has been shown to negatively affect both pregnancy outcomes and maternal oral health. However, stress is a multifactorial concept, and despite the existence of pregnancy-specific stress scales, there is no universally accepted method for its measurement [19, 20].

Therefore, the aim of this study was to evaluate the oral changes occurring during pregnancy, including soft and hard tissue alterations, salivary modifications such as cortisol levels and pH changes, and its association with maternal stress levels. Additionally, we sought to explore possible correlations among these variables, aiming to better explain the incidence of oral changes during pregnancy.

## Methods

### Study design

This study was designed as an observational cross-sectional study, aiming to study the prevalence of oral changes in pregnant (in each of the three trimesters) versus non-pregnant females, in correlation to salivary cortisol level and perceived stress level. The cross-sectional approach allows for identification of associations between oral health and biochemical/stress markers but does not establish causal relationships.

### Sample size

A power analysis was conducted to have adequate power to apply statistical test of the null hypothesis that there is no difference would be found between different tested groups regarding prevalence of oral lesions in different trimesters of pregnancy. By adopting an alpha level of (0.05) a beta of (0.2) i.e. power = 80% and an effect size (W) of (0.283) calculated based on the results of a previous study. The predicted sample size (n) was a total of (136) cases (i.e. 34 cases per group), which we later increased to 40 participants per group. All 160 participants enrolled in the study were included in the final analysis, and no missing data or attrition were recorded. Sample size calculation was performed using G*Power version 3.1.9.7 [21].

### Ethical considerations

The proposal was reviewed by the faculty of Dentistry, Ain Shams University Research Ethics Committee (FDASU-REC), research code number 1204. Written informed consent was obtained from all participants. Salivary samples have not been used for any other purposes and individual patient’s results have been kept confidential.

### Participants

A clinic-based convenience sampling strategy was used. Eligible participants were recruited consecutively from the Obstetrics and Gynecology Outpatient Clinic of the Faculty of Medicine and the Dental Diagnosis Outpatient Clinic at the Faculty of Dentistry at Ain Shams University in Cairo, Egypt during the study period until the predetermined sample size for each group was reached. Between January and June of 2024, volunteer pregnant women in different trimesters (for study groups) and non-pregnant women (for the control group) aged 18–35 years were included in this study. Pregnant participants were further divided into 3 groups according to their gestational age, each group contained participants in different trimesters of pregnancy, first (1–12 weeks), second (13–26), and third (27–40). After the patients were briefly informed about the study face to face and verbally, those who agreed to participate were included and signed a written informed consent. The current study included women with first time and previous pregnancies and a control group of non-pregnant females that were at least 15 months post-partum or postabortion. While those with any systemic disease, smokers or with history of smoking, alcohol consumption or prolonged antibiotic intake were excluded. Moreover, females that were menstruating at the time of diagnosis were excluded from the control group to avoid the hormonal fluctuations and their effect on the oral health.

### Data collection

#### Oral health questionnaires and screening

Demographic data (age, gestational age, parity), dietary changes, vomiting history, oral hygiene practices, and dental care–seeking behavior were self-reported by participants who met the eligibility criteria using a structured questionnaire. Oral health variables were assessed clinically using a standardized examination chart adapted from the World Health Organization (WHO) Oral Health Assessment Form for Adults (2013). Gingival status was evaluated based on clinical signs of inflammation, including bleeding on probing, edema, redness, and recession. Dental caries and erosion were identified by visual and tactile examination and recorded according to severity and affected surfaces. Oral soft tissue changes were defined as any clinically observable mucosal alterations, including oral candidiasis, pyogenic granuloma, aphthous ulcers, and trauma-related lesions. Each lesion was recorded according to type and location, based on standard clinical diagnostic criteria, to ensure consistent and reproducible reporting. 

#### Saliva collection for biochemical assessment

Saliva collection was done by spitting or drooling into a sterile glass tube container to collect 3 milliliters of saliva. Salivary samples for cortisol measurement were collected between 11 A.M and 1 P.M, to standardize diurnal variation while ensuring participant comfort. Participants were instructed to refrain from toothbrushing, eating, or drinking (except water) for one hour before sample collection. The sample was then transferred to the lab in storage of 4–8 degrees Celsius. Salivary cortisol concentration was expressed in ng/mL and measured using a competitive enzyme-linked immunosorbent assay (ELISA) kit (Cortisol Saliva ELISA, Cat. No. EL20-1250; Monocent, Inc., Chatsworth, CA, USA). The assay is based on a competitive immunoenzymatic colorimetric method, in which cortisol in the saliva sample competes with horseradish peroxidase-labeled cortisol for binding to anti-cortisol antibodies coated on the microplate. The kit is validated for human saliva samples and has a measurement range of 0.5–100 ng/mL, with an analytical sensitivity of 0.12 ng/mL. Absorbance was read at 450 nm with a reference wavelength of 620–630 nm, according to the manufacturer’s instructions. 

#### Stress level assessment

Perceived stress was measured using the Tilburg Pregnancy Distress Scale (TPDS), a validated questionnaire for assessing pregnancy-related stress, with higher scores indicating greater distress [22].

### Statistical methods

Categorical data were presented as frequency and percentage values and analyzed using the chi-square test, followed by pair-wise comparisons using multiple z-tests. Numerical data were presented as mean, standard deviation (SD), median, and inter-quartile range (IQR) values. Data were explored for normality by inspecting distribution patterns and using the Shapiro–Wilk test. Parametric data (age) were analyzed using an independent t-test. Other non-parametric data were analyzed using the Kruskal–Wallis test followed by Dunn’s post hoc test for multiple comparisons. P-values were adjusted for multiple comparisons using the False Discovery Rate (FDR) method. Correlation analysis was performed using Spearman’s rank-order correlation coefficient. The significance level was set at *p* < 0.05 for all tests. Statistical analysis was performed using R statistical software version 4.4.2 for Windows.

Potential confounding variables were addressed primarily through study design, including restriction of participant age, predefined inclusion and exclusion criteria, and stratification of pregnant participants by trimester. Statistical tests were selected based on variable type and data distribution, with non-parametric methods applied when normality assumptions were not met. Associations were explored using Spearman’s rank-order correlation, with correlation coefficients reported as measures of effect size to support interpretation of the findings.

## Results

Demographic data, vomiting, dietary changes, dental chief complaint, and attitude towards seeking dental treatment during pregnancy are represented in Table (1) as intergroup comparisons. Frequency of pregnancy related vomiting and whether any diet changes are present (cravings towards certain types of food, salty or sugary) were also recorded. Most pregnant females previously had children, with no significant differences between groups (*p* = 0.367). There was a significant difference between pregnant groups regarding vomiting, with a significantly higher percentage of the 1st trimester group reporting more persistent vomiting (*p* = 0.044). There was no significant difference between pregnant groups regarding dietary changes (*p* = 0.445). There was a significant difference between the tested groups regarding the chief complaint, with the percentage of 1st trimester cases reporting no complaint being significantly higher than that of the controls and those in the 3rd trimester (*p* < 0.001). Additionally, the percentage of controls complaining of pain was significantly higher than in the pregnant groups (*p* < 0.001). Finally, the percentage of cases with bleeding gums in the 2nd and 3rd trimesters was significantly higher than in the controls (*p* < 0.001). Most of the study group with a percentage of 76.6% reported unwillingness to seek dental treatment during pregnancy.

**Table 1:** Intergroup comparisons and summary statistics for demographic data, vomiting history, diet changes, dental chief complaint and seeking medical treatment

Parameter		1st trimester	2nd trimester	3rd trimester	Controls	*p*-value
Age (years)	Mean±SD	28.62±4.61	28.40±4.96	28.92±5.43	27.35±5.22	0.535ns 1
	Median (IQR)	29.50 (7.25)	28.00 (9.00)	30.00 (9.25)	28.00 (8.25)	
Parity	Nulliparous	33 (82.50%)	28 (70.00%)	32 (80.00%)	NA	0.367ns2
	Primi/multiparous	7 (17.50%)	12 (30.00%)	8 (20.00%)	NA	
Vomiting [n (%)]	No	21 (52.50%)	19 (47.50%)	20 (50.00%)	NA	0.044
	Yes	2 (5.00%)	12 (30.00%)	9 (22.50%)	NA	
	Persistent	17 (42.50%)	9 (22.50%)	(27.50%)	NA	
Dietary changes [n (%)]	No changes	18 (45.00%)	16 (40.00%)	23 (57.50%)	NA	0.445ns2
	Higher salty food intake	10 (25.00%)	8 (20.00%)	5 (12.50%)	NA	
	Higher sugar intake	12 (30.00%)	16 (40.00%)	12 (30.00%)	NA	
Dental chiefcomplain[n (%)]	No dental chief complain	20 (50.00%)	13 (32.50%)	9 (22.50%)	7 (17.50%)	<0.001
	Pain	7 (17.50%)	7 (17.50%)	11 (27.50%)	27 (67.50%)	
	Bleeding gums	13 (32.50%)	20 (50.00%)	20 (50.00%)	6 (15.00%)	
Seeking dental treatment during pregnancy [n (%)]	No	29 (72.50%)	29 (72.50%)	34 (85.00%)	NA	0.477ns2
	Yes	8 (20.00%)	6 (15.00%)	3 (7.50%)	NA	
	Only in emergency	3 (7.50%)	5 (12.50%)	3 (7.50%)	NA	

1= One-way ANOVA, 2 = Chi-squared test

### Findings of comprehensive intra and extraoral examination

**Fig. 1:**
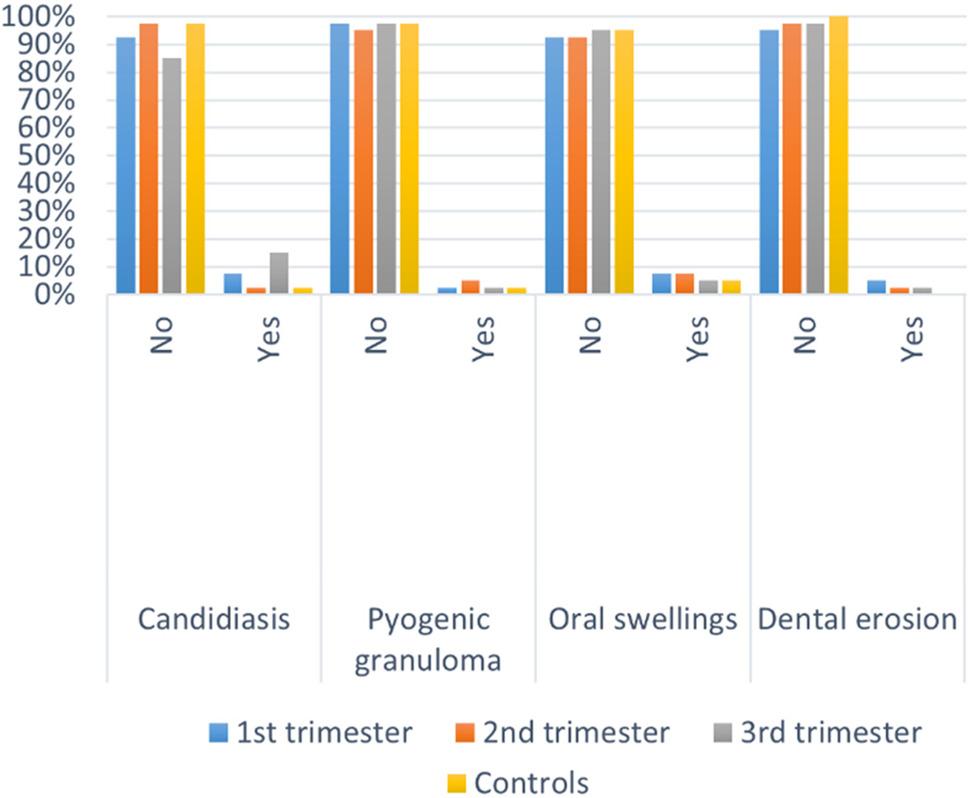
Bar chart showing parameters of oral examination and history. Test = Chi-squared test

Intergroup comparisons and summary statistics for the results of dental examination are presented in Tables (2, 3) and Fig. (1). There was a statistically significant difference regarding gingival health, with the percentage of cases with healthy gingiva in the control group being significantly higher than the 3rd trimester (*p* < 0.001). In addition, the percentage of cases in the 1st, 2nd and 3rd trimesters affected with gingival changes (diseased gingiva) was significantly higher than the controls (*p* < 0.001). The percentage of caries incidence was higher in the control group than any of the three trimesters but with no statistically significant difference (*p* > 0.05). The incidence of soft tissue lesions was equal in all three trimesters of the study group and higher than the control group but with no statistically significant difference. The overall incidence of candidiasis, oral swellings, pyogenic granuloma and dental erosion was found to be higher in the study group (all three trimesters combined) than the control group.

**Table 2:** Intergroup comparisons and summary statistics for the results of oral examination

Parameter		1st trimester	2nd trimester	3rd trimester	Controls	*p*-value
Soft tissue lesions [n (%)]	No	28 (70.00%)	28 (70.00%)	28 (70.00%)	33 (82.50%)	0.496ns1
	Yes	12 (30.00%)	12 (30.00%)	12 (30.00%)	7 (17.50%)	

1 = Chi-squared test

**Table 3:** Intergroup comparisons and summary statistics for the results of oral examination

Gingival health	n (%)				*p*-value
	1st trimester	2nd trimester	3rd trimester	Controls	
Healthy	12 (30.00%)	9 (22.50%)	6 (15.00%)	17 (42.50%)	0.041
Not healthy	28 (70.00%)	31 (77.50%)	34 (85.00%)	23 (57.50%)	

1 = Chi-squared test

### Results for salivary cortisol and pH

**Fig. 2:**
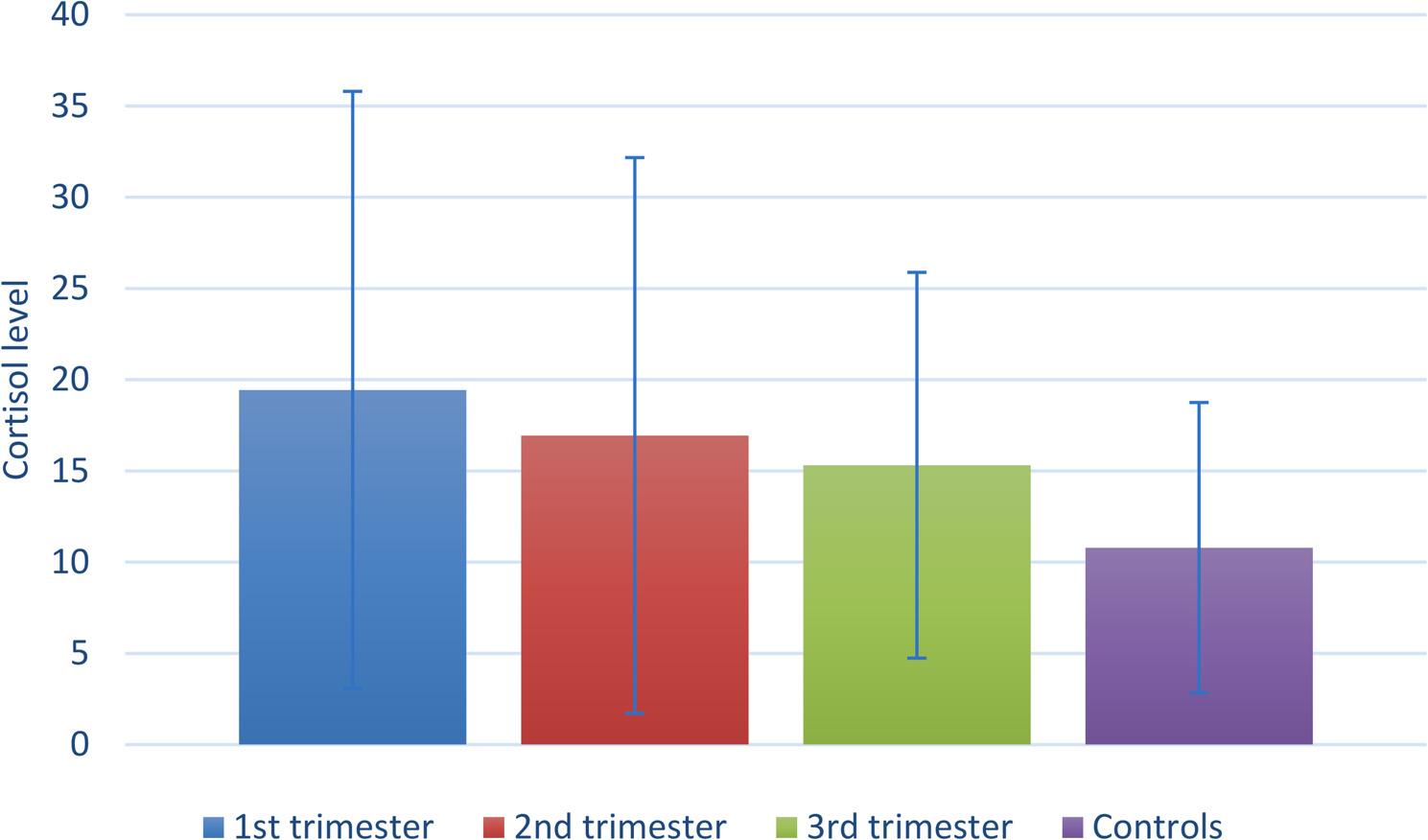
Bar chart showing mean and standard deviation values ofcortisol level. Test = Kruskal-Wallis test, *p* = 0.044

Intergroup comparisons and summary statistics for salivary biochemical assessment are presented in Table (4) and Fig. (2). There was a significant difference between tested groups regarding levels of salivary pH, with the control group having significantly higher values than 1st and 2nd trimesters (*p* < 0.001). Similarly, the difference was also statistically significant for cortisol levels, with the 1st trimester group having significantly higher values than the controls (*p* = 0.044).

**Table 4:** Intergroup comparisons and summary statistics for biochemical assessment

Parameter		1st trimester	2nd trimester	3rd trimester	Controls	*p*-value
Salivary pH	Mean±SD	6.19±0.37	6.12±0.44	6.31±0.50	7.15±2.38	<0.001
	Median (IQR)	6.10 (0.54)	6.00 (0.72)	6.36 (0.66)	6.55 (0.90)	

1= Kruskal-Wallis test

### Results of stress analysis using the TPDS

Intergroup comparisons and summary statistics for stress analysis are presented in Table (5). The TPDS was slightly higher in the first trimester than the second and third trimesters but there was no significant difference between different groups.

**Table 5:** Intergroup comparisons and summary statistics for stress analysis

Parameter		1st trimester	2nd trimester	3rd trimester	Controls	*p*-value
TDPS	Mean±SD	19.10±6.24	18.20±5.35	18.15±5.39	NA	0.799ns1
	Median (IQR)	19.00 (10.25)	17.00 (7.25)	17.50 (8.00)	NA	

1= Kruskal-Wallis test

**Fig. 3:**
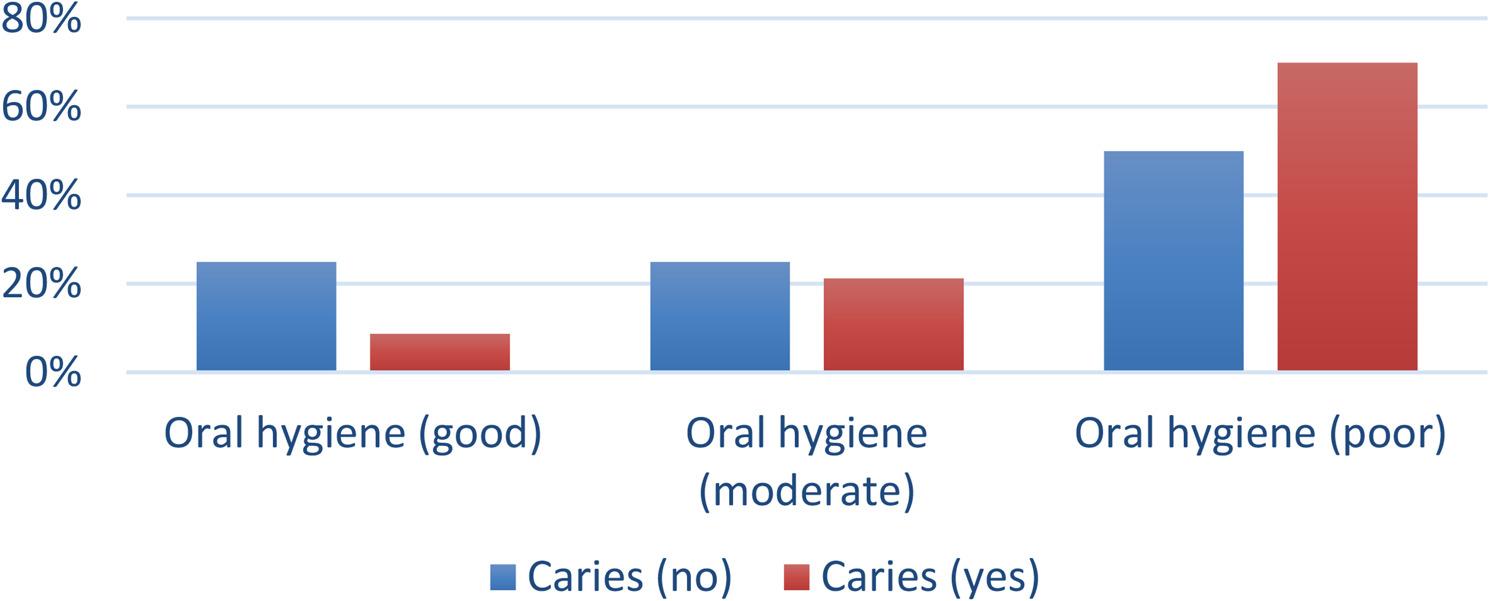
Bar chart showing the association between oral hygiene level and caries incidence. Test = Chi-square test, *p* = 0.033

**Fig. 4:**
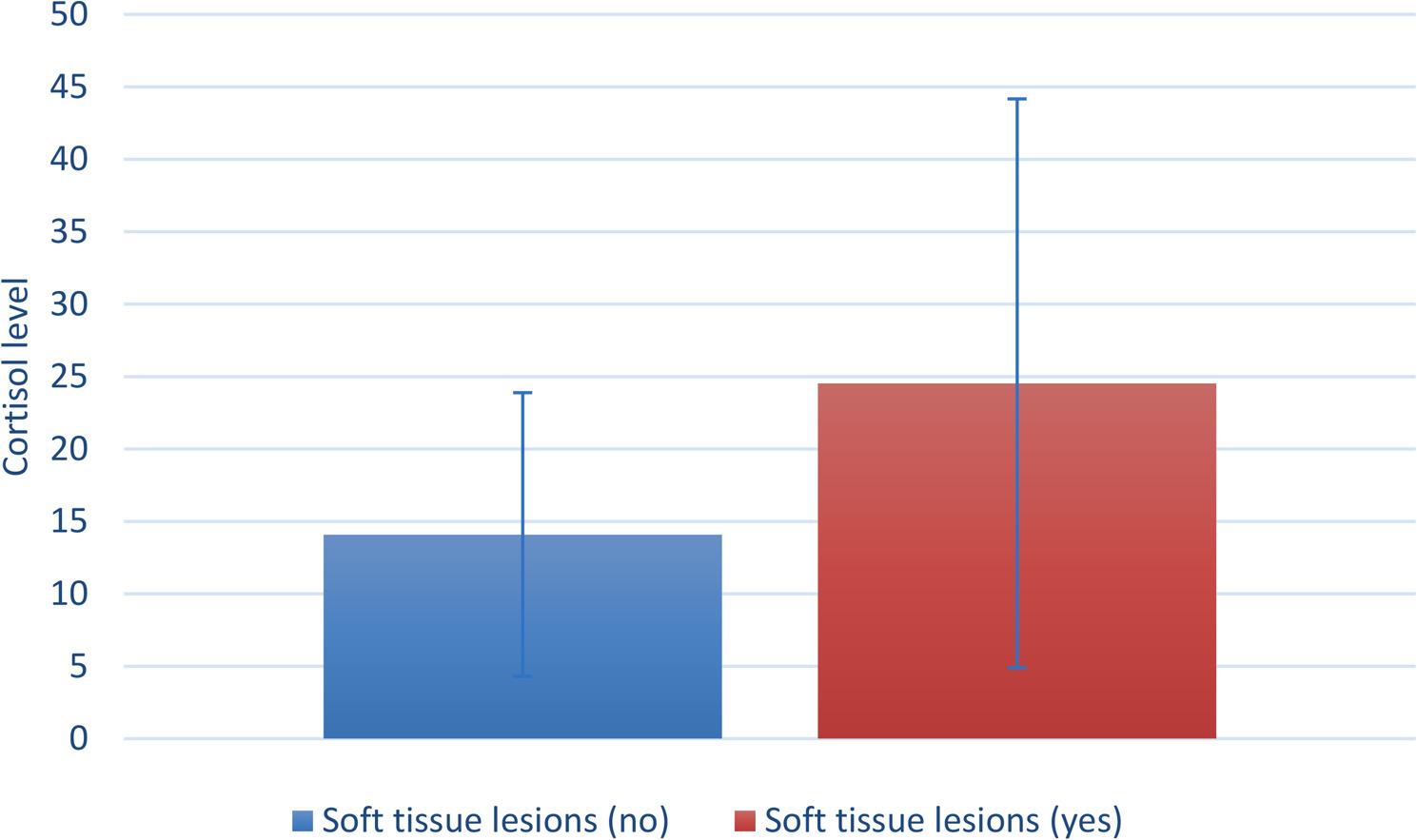
Bar chart showing the association between salivary cortisol and incidence of soft tissue lesions. Test = Kruskal-Wallis test, *p* = 0.019

**Fig. 5:**
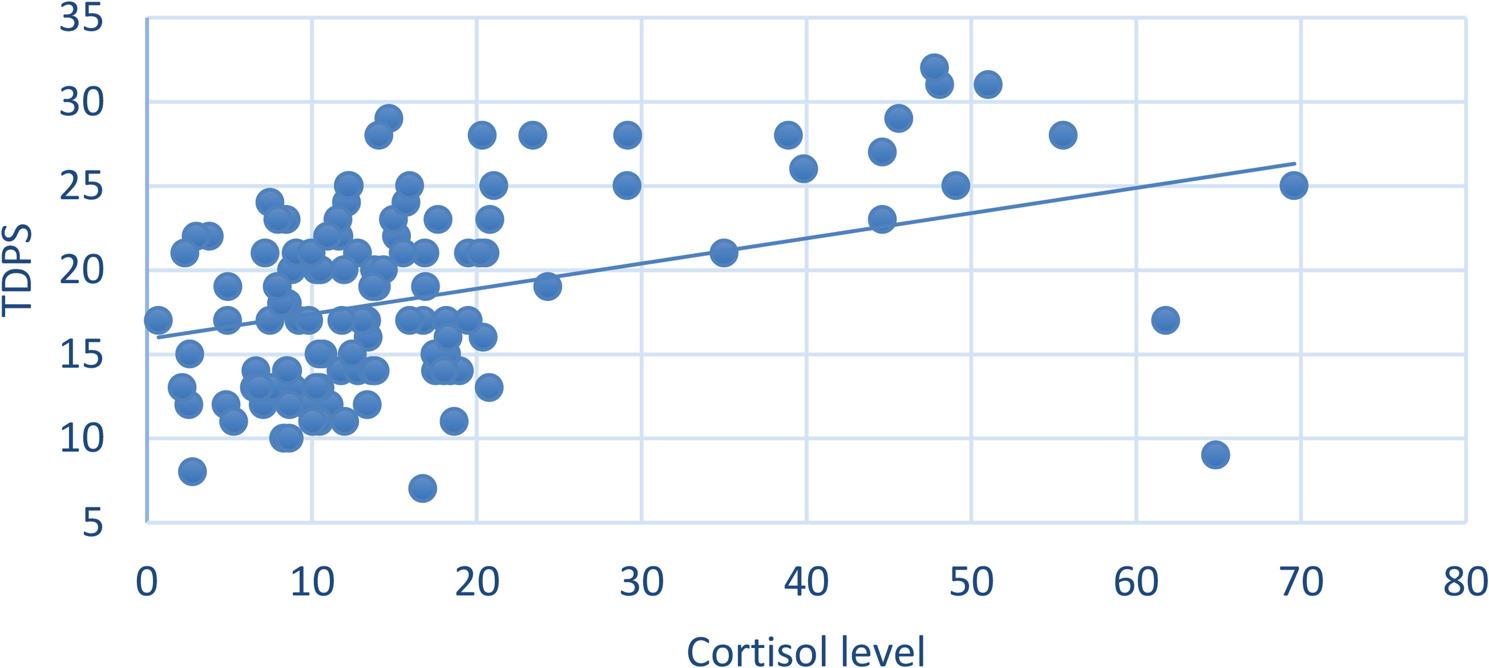
Scatter plot showing the correlation between TDPS score and salivary cortisol. Test = Spearman’s correlation coefficient, *ρ* = 0.392, *p* < 0.001

Correlations between different variables from previous results among the study groups:

- The association between the level of oral hygiene and caries incidence is presented in Fig. (3). The association was statistically significant, with pregnant cases with good oral hygiene having significantly lower incidences of caries and cases with poor hygiene having significantly higher incidences (*p* = 0.033).
- 50% of the cases with oral candidiasis suffered persistent vomiting during pregnancy, however, the association was not statistically significant. (Table (6)).
- The current results showed that 100% of the patients with dental erosion suffered from vomiting during gestation but the association was not statistically significant.
- The association between the incidence of soft tissue lesions and salivary cortisol level was statistically significant, with affected cases having significantly higher cortisol levels (*p* = 0.019) (Fig. 4).
- Another correlation observed in the present study was the relation between salivary cortisol and gingival health. There was a positive correlation between gingival condition and cortisol level.
- It was found that patients representing signs of oral soft tissue changes had higher TPDS though the association was not statistically significant (*p* = 0.336).
- Finally, we attempted to correlate stress levels measured by TPDS in the study group with levels of salivary cortisol and a positive correlation was observed, which was statistically significant (*p* < 0.001) Fig. (5).

**Table 6:** Association between vomiting and the presence of candidiasis

Vomiting	Candidiasis		*p*-value
	No	Yes	
No	56 (50.91%)	4 (40.00%)	0.369ns1
Yes	22 (20.00%)	1 (10.00%)	
Persistent	32 (29.09%)	5 (50.00%)	

1 = Chi-squared test.

## Discussion

A woman’s pregnancy is a special time in her life. Pregnancy causes several complex physiological changes in the body, which may be accompanied by changes in oral health. Pregnant women’s dental health care demands differ greatly from those of the general population, according to previous research [23]. In addition, widespread myths and misconceptions regarding dental care during pregnancy may limit appropriate utilization of oral health services, underscoring the importance of effective communication between oral health and prenatal care providers. Consequently, it’s important to better understand pregnant women’s dental health and behavior.

A recent systematic review published in 2024 by *Pecci-Lloret* covered the topic of oral manifestations in pregnant women and they concluded that many more studies are needed on the same issue to encounter the limitations that they faced. In this context, the present cross‑sectional study provides region‑specific data on oral changes observed during pregnancy in an Egyptian population and explores their associations with salivary cortisol levels and pregnancy‑related stress. While several oral, periodontal, and salivary alterations were observed among pregnant participants, the study design allows only for the identification of associations and not for determination of causality [24].

Effective screening is essential to help reduce the prevalence of oral disease and assess the oral health status of pregnant females. Therefore, the primary aim of the present study was to estimate the prevalence of oral lesions and salivary changes in a sample of pregnant Egyptian females.

To minimize potential confounding factors, pregnant women above 35 years old were excluded to reduce the potential influence of advanced maternal age can pose on the results [25]. In addition, the control group comprised non-pregnant females who were at least 15 months after the last pregnancy or abortion to help minimize any residual effects of pregnancy-related changes on oral health [26].

For clarity, the results of this study were classified into 4 sections: demographic data, oral examination findings, biochemical assessment results and stress analysis results.

### Demographic data

Regarding demographic characteristics, data included age, parity, vomiting history, diet changes, and dental chief complaint. The history of vomiting during pregnancy was reported by 50% of the participants in the study group, with a significantly higher percentage of the 1st trimester group reporting more persistent vomiting (defined as vomiting that started early in pregnancy and had not resolved, with fewer than five episodes per day), consistent with earlier studies [27, 28]. Dietary changes were also evaluated revealing that 47.55% of the study group showed no specific food cravings, while 33.3% reported higher sugar craving and intake, and 19.16% reported cravings for salty food, however, these differences were not statistically significant. These findings are consistent with previous studies reporting that food cravings are common during pregnancy, with a tendency for sweets and salty foods to be most frequently desired, reflecting typical gestational dietary patterns [29].

Dental chief complaints were more frequently reported by the control (82.5%), compared with pregnant participants across the first (50%), second (67.5%) and third (77.5%) trimesters, with statistically significant difference. These findings also differ from a study in Utah, USA, which reported only 26% of pregnant women requiring dental care, possibly reflecting differences in socioeconomic status, oral health awareness, and access to dental services [30]. This discrepancy may be related to the different socioeconomic state, oral health care and educational level of females in Egypt. Furthermore, the higher prevalence of dental complaints in the control group may be partly attributable to recruitment from a dental outpatient clinic, whereas pregnant participants were recruited from an obstetrics and gynecology setting.

Behavioral patterns regarding dental care utilization during pregnancy revealed substantial reluctance to seek treatment. In the present study, 76.6% of pregnant participants declined dental treatment during gestation despite experiencing dental pain, while only 14.1% reported willingness to seek dental care if needed, and 9.1% would do so only in emergency situations, with no statistically significant difference. These findings are consistent with reports from other regions and may reflect persistent misconceptions, concerns regarding maternal and fetal safety, financial and time constraints, and limited referral from healthcare providers [31].

### Oral examination findings

Intra-oral examination findings covered gingival health, soft tissue lesions, caries and dental erosion. Clinical oral examination revealed a high prevalence of periodontal and gingival changes which were statistically significant, affecting 77.5% of pregnant participants across all trimesters. Among these, 63.3% exhibited signs of gingival disease, including bleeding on probing, edema, redness, and recession. These findings align with a 2024 systematic review and a study conducted in the UAE reporting gingivitis prevalence of 71.9% among pregnant women [11, 25]. In contrast, lower prevalence (45.8%) has been documented in Japan, which may reflect differences in oral hygiene practices, socioeconomic status, and healthcare access [32].

Dental caries prevalence was also high, with 66.6% of pregnant participants exhibiting signs of decay. This may be associated with increased carbohydrate intake, vomiting-induced acid exposure, reduced salivary flow, and suboptimal oral hygiene practices. These figures are similar to Velosa-Porras et al. (59%) and Patil et al. (63.3%). The observed prevalence highlights the importance of preventive interventions tailored to socioeconomic and cultural contexts [11, 33].

An additional association was observed in the current study between parity and tooth loss. Participants experiencing their first pregnancy exhibited minimal tooth loss, whereas those with multiple full-term pregnancies demonstrated higher numbers of missing teeth, primarily due to caries or mobility. This comes in agreement with the results published by *Wandera et al.* and Russell et al. but this relation couldn’t be proved by *Ueno et al.*in Japan [34–36].

Oral mucosal lesions were identified in 30% of pregnant participants, evenly distributed across trimesters, compared with 17.5% in the control group, with no statistically significant difference between groups. These results align with Díaz Guzmán et al. in Mexico (30%) but exceed population-based studies by Bett et al. (16.5%) and Duarte da Silva et al. (11.8**%)** which may be influenced by differences in socioeconomic status, oral hygiene practices, and ethnicity [37–39].

The increased prevalence of oral mucosal lesions observed during pregnancy has been suggested in the literature to be multifactorial, involving elevated estrogen and progesterone levels, increased vascular permeability, immunological modulation, pregnancy-related stress, and nutritional changes [13, 40]. In the present study, oral candidiasis was the most prevalent lesion (8.3%), exceeding rates reported in previous systematic reviews [38]. Pyogenic granuloma was observed less frequently (3.3%), with a higher proportion noted during the second trimester, consistent with previously reported hormonal associations. However, both findings did not demonstrate statistically significant differences between groups.

Dental erosion was identified in 3.3% of pregnant participants, with higher prevalence in the first trimester (5%), which may be related to vomiting and acid reflux that cause exposure of tooth surfaces, but the result was not statistically significant [2]. Although dental erosion prevalence varies among populations, prior studies have reported similarly low incidence rates in pregnant women, with some cohorts showing prevalence as low as 1–2%, highlighting the variability of erosion patterns depending on dietary habits, vomiting frequency, and regional differences [41].

### Biochemical assessment

Salivary assessment included measurement of salivary pH and salivary cortisol. In the present study, salivary pH was found to be significantly lower in the 1st and 2nd trimesters, which may be related to the reduction in salivary pH that occurs during gastro-esophageal reflux, as well as potential hormonal influences such as progesterone-related changes in plasma bicarbonate levels. During pregnancy, reduced salivary pH may may also be related to changes in taste, increased eating frequency, and variations in oral hygiene practices [16]. This observation is in line with previous reports indicating that reduced salivary pH during pregnancy is associated with an increased risk of oral disease, including erosion and soft tissue lesions, emphasizing the impact of pregnancy-related physiological changes on oral health [21].

Salivary cortisol levels can represent serum cortisol levels with 80% accuracy; therefore, the current study measured morning salivary cortisol levels and concluded a significant increase in each of the three trimesters of pregnancy when compared to the control group. The mean salivary cortisol was higher in the first trimester (19.44 ng/ml), than the second (16.94 ng/ml) and third (15.31 ng/ml). This comes in agreement with a study by *Schowe et al.*, in which the cortisol awakening response slope declined from early to late pregnancy. However, this comes in opposition to previously published results by *Allolio et al.*and Obel et al. in which the levels in late pregnancy were significantly higher than in early pregnancy [42–45].

### Stress assessment

Stress analysis measured using TPDS showed that the scores were higher in the first trimester than the second and the third, which may correspond with the observed pattern of higher salivary cortisol levels in early pregnancy. The TDPS scores should no statistically significant differences.

### Observed associations between variables

Different associations were identified between oral findings and biochemical or behavioral factors. An association was found between the level of oral hygiene and caries incidence, in the study group, where 70% of the participants with caries had poor oral hygiene, while those with good oral hygiene had only an 8.75% incidence of caries, and those results were statistically significant. This could be confirmed with a study conducted by Martínez Nieto et al., in which improving personalized oral hygiene was associated with better oral health outcomes and lower caries prevalence [46].

Vomiting has been suggested as a potential contributing factor to oral candidiasis during pregnancy by possibly creating favorable conditions such as disruption of oral pH, mucosal irritation, and xerostomia secondary to dehydration. In the present study, 50% of participants with oral candidiasis reported persistent vomiting during pregnancy; however, this association did not reach statistical significance [47].

Several studies have reported an association between higher salivary cortisol level and many oral soft tissue lesions (recurrent aphthous ulcer, oral lichen planus, and oral potentially malignant disorders). Such an association was found to be statistically significant in the current study, where patients with various oral soft tissue lesions had significantly higher salivary cortisol levels [48–50].

Another association was observed in the current study between salivary cortisol and gingival health, where the participants with affected gingiva showed higher mean of salivary cortisol than those with healthy periodontium, though it wasn’t statistically significant. These results came in accordance with a study performed by *Lee et al.* on a different study sample (males and non-pregnant females), which suggested that the level of salivary cortisol may be associated with increased severity of periodontal disease. The same study also mentioned the possible cause as increased cortisol levels can change the makeup of saliva, decreasing its antibacterial and buffering capabilities, which could potentially contribute to an environment favorable for pathogenic bacterial growth and gingival inflammation [51].

Lower salivary pH was observed among participants with dental erosion, although this association did not reach statistical significance.

Finally, higher psychosocial stress scores were observed among participants presenting with oral soft tissue changes, suggesting a possible association between stress and oral mucosal alterations. Psychosocial stress was assessed using the TPDS, and participants with oral soft tissue changes demonstrated higher TPDS scores; however, this association was not statistically significant. In contrast, a statistically significant positive association was identified between TPDS scores and mean salivary cortisol levels, supporting the relationship between perceived stress and biochemical stress markers [51, 52]. The study has several limitations that should be acknowledged. First, the relatively small study sample size and its localization to one center may restrict the generalizability of the finding. Second, the cross-sectional design precludes inference of causal relationships between pregnancy, oral changes, and stress biomarkers. Third some data relied on self-reported information, which may introduce recall or reporting bias. Fourth, the clinic-based recruitment strategy may have introduced selection bias, and all clinical examinations were conducted by a single examiner, which could contribute to observer bias. Fifth, the control group was recruited from a dental outpatient clinic, which may have led to selection bias and an overestimation of dental complaints compared to the general population; this should be considered when interpreting the findings. Finally, certain diagnostic tools, such as full periodontal charts and radiographs, were not feasible due to ethical and practical constraints in the study population. Despite these limitations, the study provides valuable insights into pregnancy-associated oral and salivary changes, and future multicenter longitudinal studies with larger samples are recommended to confirm and extend these findings.

## Conclusion

The current study demonstrates that pregnancy is associated with a high prevalence of gingivitis (77.5%), dental caries (66.6%), and oral mucosal lesions (30%). These findings may be related to factors such as suboptimal oral hygiene practices, hormonal changes, and salivary alterations observed during pregnancy. In addition, higher psychosocial stress levels during gestation were associated with increased salivary cortisol levels, which may be linked to the presence and severity of oral health findings.

Despite the observed burden of oral conditions, a substantial majority (76.6%) of pregnant women avoided dental treatment during pregnancy, possibly reflecting persistent misconceptions, limited awareness, and concerns regarding the safety of dental care. These findings highlight the importance of targeted educational interventions and support the integration of oral health care into routine prenatal programs.

## Data Availability

The datasets generated and/or analyzed during the current study are available from the corresponding author on reasonable request.

## Abbreviations

ELISA: Enzyme Linked Immunosorbent Assay
FDR: False Discovery Rate
HSV: Human Simplex Virus
IQR: Interquartile Range
SD: Standard Deviation
TPDS: Tilburg Pregnancy Distress Scale
WHO: World Health Organization
